# Health outcomes of bedaquiline in the treatment of multidrug-resistant tuberculosis in selected high burden countries

**DOI:** 10.1186/s12913-016-1931-3

**Published:** 2017-01-26

**Authors:** Xiaoyan Lu, Caitlin Smare, Chrispin Kambili, Antoine C. El Khoury, Lara J. Wolfson

**Affiliations:** 1 Janssen Pharmaceutica NV, Beerse, Belgium; 2 HERON™ Commercialization, PAREXEL International, London, UK; 3 Janssen Global Services LLC, Raritan, NJ USA; 4 Johnson and Johnson Middle East FZ LLC, Mohammed Bin Rashid Al Makhtoum Academic Medical Centre, Building 14, PO Box 505080, Dubai, United Arab Emirates

**Keywords:** DALY, Cost-effectiveness threshold

## Abstract

**Background:**

Less than one-third of patients who are estimated to be infected with multidrug-resistant tuberculosis (MDR-TB) receive MDR-TB treatment regimens, and only 48% of those who received treatment have successful outcomes. Despite current regimens, newer, more effective and cost-effective approaches to treatment are needed. The aim of the study was to project health outcomes and impact on healthcare resources of adding bedaquiline to the treatment regimen of MDR-TB in selected high burden countries: Estonia, Russia, South Africa, Peru, China, the Philippines, and India.

**Methods:**

This study adapted an existing Markov model to estimate the health outcomes and impact on total healthcare costs of adding bedaquiline to current MDR-TB treatment regimens. A price threshold analysis was conducted to determine the price range at which bedaquiline would be cost-effective.

**Results:**

Adding bedaquiline to the background regimen (BR) resulted in increased disability-adjusted life years (DALYs) averted, and reduced total healthcare costs (excluding treatment acquisition costs) compared with BR alone in all countries analyzed. Addition of bedaquiline to BR resulted in savings to healthcare costs compared with BR alone in all countries analyzed, with the highest impact expected in Russia (US$194 million) and South Africa (US$43 million). The price per regimen at which bedaquiline would be cost-effective ranged between US$23,904-US$203,492 in Estonia, Russia, Peru, South Africa, and China (high and upper middle-income countries) and between US$6,996-US$20,323 in the Philippines and India (lower middle-income countries); however, these cost-effective prices do not necessarily address concerns about affordability.

**Conclusions:**

Adding bedaquiline to BR provides improvements in health outcomes and reductions in healthcare costs in high MDR-TB burden countries. The range of prices per regimen for which bedaquiline would be cost-effective varied between countries.

**Electronic supplementary material:**

The online version of this article (doi:10.1186/s12913-016-1931-3) contains supplementary material, which is available to authorized users.

## Background

Multidrug-resistant tuberculosis (MDR-TB), defined as tuberculosis caused by strains resistant to isoniazid and rifampicin, poses challenges to global TB control. Extensively drug-resistant TB (XDR-TB), caused by MDR-TB strains that are also resistant to any fluoroquinolone and to at least one of the three injectable second-line drug classes (aminoglycosides, polypeptides, fluoroquinolones, thioamides, cycloserine, and para-aminosalicyclic acid) [1] may result in even poorer outcomes compared with MDR-TB [2]. In 2013, it was estimated that 3.5% of newly-diagnosed and 20.5% of previously treated TB cases had MDR-TB, and XDR-TB accounted for 9.0% of all MDR-TB cases [2]. Prompt diagnosis of MDR-TB and initiation of appropriate therapy provide the best chance of favorable treatment outcomes [3].

Bedaquiline received accelerated and conditional approval in the United States and Europe, respectively, for the treatment of pulmonary MDR-TB in combination with other anti-tuberculosis drugs when an effective treatment regimen cannot otherwise be provided [4, 5]. The World Health Organization (WHO) subsequently issued interim guidance for use of bedaquiline in adult patients with pulmonary MDR-TB [6].

A study commissioned by the WHO previously explored the cost-effectiveness (CE) of adding bedaquiline to 20 month MDR-TB treatment regimens in six low- to middle-income settings (Russia, Estonia, the Philippines, Peru, Nepal, and China) [7], covering 17% of global MDR-TB burden in 2013 [2]. The WHO study showed that although bedaquiline was likely to be cost-effective and cost-saving in Peru, Russia, Estonia, The Philippines, and China, where treatment and management costs are moderate to high, it may not be cost-effective in low-income countries with low drug costs, such as Nepal [7, 8]. Using a model with a longer time horizon [9], the current study assessed the health outcomes and impact on healthcare costs of using bedaquiline in seven countries, including China, India and South Africa, where MDR-TB has been reported to be highly prevalent; collectively, the seven countries analyzed in the current study accounted for approximately 60% of the global MDR-TB burden in 2013 [2]. Nepal was not included in the current study due to lack of available data at the time of the analysis (including data related to country-specific epidemiology, treatment pathways and healthcare costs).

### Objectives

The primary objective of the study was to evaluate health outcomes, expressed in terms of disability-adjusted life years (DALYs) averted, following addition of bedaquiline to the background regimen (BR) in the treatment of MDR-TB in an economically diverse group of high MDR-TB burden countries (Estonia, Russia, South Africa, Peru, China, the Philippines, and India) (Additional file 1: Table S1). The potential impact of adding bedaquiline on total healthcare costs was also assessed. An exploratory price analysis was conducted to estimate the likelihood of bedaquiline being cost-effective as measured by cost per DALY in each country setting, based upon thresholds provided by the WHO [6]. It was not possible to conduct a full CE analysis on the use of bedaquiline for the treatment of MDR-TB because, at the time of the analysis, there were no publicly listed prices for bedaquiline in the selected countries.

## Methods

### Model overview

A previously-described, cohort-based Markov state transition model [9] was adapted to evaluate the health and economic benefits of adding bedaquiline to a BR compared with BR alone, for the treatment of patients with MDR-TB from the healthcare system perspective of seven countries with a high MDR-TB burden: Estonia, Russia, South Africa, Peru, China, the Philippines, and India [2].

Health outcomes and healthcare costs were simulated over a 10-year time horizon to capture downstream consequences of treatment. The effectiveness of treatment was evaluated in terms of DALYs averted. Definitions of clinical and outcome data were sourced from the literature [10, 11].

### Model structure

**Fig. 1:**
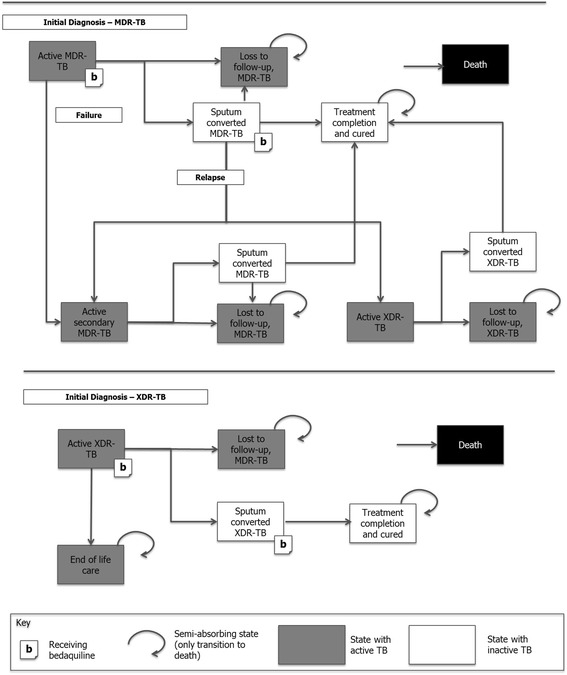
Outline of the Markov model assessing health outcomes of bedaquiline in high burden countries Source: adapted from [9] CE: cost-effectiveness; MDR-TB: multidrug-resistant tuberculosis; XDR-TB: extensively drug-resistant tuberculosis. Note: MDR-TB population includes patients with MDR-TB as well as XDR-TB patients; transitions to the death state are possible from every state, but not shown on the diagram for clarity

The analysis included new laboratory-confirmed cases of MDR-TB from each of the selected high MDR-TB burden countries. Details of the model structure are described in Wolfson et al*.* [9]. In brief, patients entered the model in the “Active MDR-TB” or “Active XDR-TB” states, depending on their level of resistance and would experience slightly different clinical pathways (Fig. 1). Patients could transition in 28-day cycles in the model through health states such as sputum culture conversion, treatment completion, loss to follow-up, and death. The aim of treatment was to achieve sputum culture conversion (transition to sputum culture converted MDR-TB), and maintain the converted state until treatment completion and assumed cure of MDR-TB (transition to treatment completion). All patients who culture converted would transition to the cured health state until they had completed treatment or relapsed. Once treatment would be completed, patients would transition to the completed and cured health state. Patients faced different mortality risks depending on the status of their culture conversion (1.22% before culture conversion, 0.18% following culture conversion, and all-cause mortality once they completed treatment and were cured).

Treatment failures in the model consisted of patients who relapsed (defined as having a positive sputum culture after achieving sputum culture conversion) [12, 13], had a recurrence of TB (defined as developing active TB after treatment completion), or remained sputum culture positive after 1 year of treatment. Patients with MDR-TB who failed treatment or relapsed could develop additional resistance and experience XDR-TB.

Patients could become lost to follow-up (default) anytime while on treatment. Patients initially diagnosed with XDR-TB (‘Active XDR-TB’ state) could experience similar outcomes (relative disability weight of 1.20 for XDR-TB) to those initially diagnosed with MDR-TB except that no subsequent treatment was allowed due to the limited treatment options in this more severe patient group. Patients with XDR-TB who failed treatment transitioned to end of life care (palliative care), where they experienced higher mortality and worse health outcomes.

### Clinical data

Data on the efficacy of treatment and the risk of morbidity and mortality in patients with MDR-TB were obtained from various sources, including the C208 study - a Phase II, placebo-controlled, randomized trial of bedaquiline in newly diagnosed MDR-TB patients [12, 13]. In addition, country-specific outcomes were also used for the analysis [14, 15].

Comparative data were sourced in the form of hazard ratios (HRs) from published clinical trial data [13] (Table 1) and applied to data for patients receiving BR only to estimate relative treatment effect.

**Table 1:** Disease transition probabilities for the high burden countries analyzed

Parameter			Probability of event/28 days, % (SE)		[Source]		
Sputum culture conversion on BR, 0–8 weeks (log-normal)							
Scale parameter			4.99 (0.21)		Placebo-controlled phase II clinical trial C208 [12]		
Shape parameter			0.73 (0.11)		Placebo-controlled phase II clinical trial C208 [12]		
Sputum culture conversion on BR, 8–24 weeks (log-normal)							
Scale parameter			5.68 (0.40)		Placebo-controlled phase II clinical trial C208 [12]		
Shape parameter			1.90 (0.27)		Placebo-controlled phase II clinical trial C208 [12]		
Sputum culture conversion on BR, ≥24 weeks (log-normal)							
Scale parameter			8.28 (1.09)		Placebo-controlled phase II clinical trial C208 [12]		
Shape parameter			2.70 (0.82)		Placebo-controlled phase II clinical trial C208 [12]		
Subsequent MDR-TB							
Hazard ratio of subsequent MDR-TB (vs. initial MDR-TB)			0.54 (0.17)		Open-label, phase II clinical trial C209 [13]		
Other events							
Probability of reversion			1 (0.30)		Placebo-controlled phase II clinical trial C208 [12]		
Probability of death	MDR-TB, no cure		2.21		[34, 35]		
	MDR-TB, cure		0.32		[34, 35]		
	XDR-TB, no cure		2.69		[34, 35]		
	XDR-TB, cure		0.39		[34, 35]		
Treatment effect							
Hazard ratio of bedaquiline (sputum culture conversion)			2.44 (0.57)		Placebo-controlled phase II clinical trial C208 [12]		
Hazard ratio of bedaquiline (relapse)			0.32 (0.25)		Placebo-controlled phase II clinical trial C208 [12]		
Hazard ratio (XDR-TB)			0.40 (0.11)		Open-label, phase II clinical trial C209 [13]		
	Estonia^a^	Russia^b^	South Africa^a^	Peru^a^	China^a^	Philippines^a^	India^a^
Probability of loss to follow-up, % (SE)	1.4 (4.1)	1.5 (0.3)	1.4 (0.4)	1.5 (0.8)	0.6 (0.7)	3.4 (1.6)	1.2 (0.6)

SOURCE: (^a^): [14]; (^b^): [15]

*BR* background regimen, *MDR-TB* multidrug-resistant tuberculosis, *SE* standard error, *XDR-TB* extensively drug-resistant tuberculosis

Rates of sputum culture conversion for patients receiving BR treatment alone were estimated from a post-hoc patient-level analysis of published clinical data from the C208 study [12, 13]. The cumulative probability of remaining sputum culture positive was split into three time periods (<8 weeks, 8–24 weeks, and >24 weeks), based on the best fit of survival probabilities observed in the placebo arm of a bedaquiline clinical trial [13]. A log-normal distribution was fitted to each period to derive rates for the entire model time horizon (Table 1).

The HR on sputum culture conversion for bedaquiline compared with BR was applied for the duration of bedaquiline treatment (i.e. 24 weeks), after which patients were assumed to experience no further reduction in the rate of sputum culture conversion. The HR on sputum culture conversion for patients with XDR-TB was generated based on proxy data from patients with pre-XDR-TB collected in an open-label bedaquiline study [13]; this was applied onto the time-to-sputum culture conversion curves to estimate the sputum culture conversion rates for XDR-TB patients.

### Outcomes data

Disability adjusted life years (DALY), and years of life lost due to premature mortality were sourced from two sources: a global burden of disease study that reported disability weights for active TB and the UK life tables [16, 17]. The disability weight for treatment completion was assumed to be zero (i.e. no disability).

### Cost data

Cost data were sourced from country-specific databases [18–23], hospitals, and scientific opinion leaders, as well as published literature [8, 24–26]. Direct medical costs, treatment monitoring costs, and administered care (hospitalization and outpatient) costs were included in the model (Table 2). It was assumed that 100% of patients were hospitalized until sputum culture conversion (except for Estonia, India, and South Africa, where 80%, 5%, and 10%, respectively, of patients with MDR-TB were hospitalized with those not hospitalized assumed to have received outpatient care), after which they would receive outpatient care only. Treatment acquisition costs for bedaquiline were excluded from the cost analysis since the price was unavailable in the countries evaluated at the time of this analysis. The preferred BR regimen for this analysis included ethionamide, ofloxacin, pyrazinamide, ethambutol, and cycloserine, based on the C208 study [12]. The cost of monitoring patients while on treatment was dependent on time spent on each treatment, the phase of treatment (intensive vs. continuous), and the range of clinical tests required. Costs were sourced from publically available country-specific tariffs (Table 2). Both costs and benefits were discounted at annual rates recommended by local guidelines (China: 6.0%; Peru: 3.0%; and Estonia, Russia, South Africa, India, and the Philippines: 5.0%) [27–29].

**Table 2:** Cost and disability weight inputs for the high burden countries analyzed, US$ 2013^a^

Parameter		Country						
		Estonia	Russia	South Africa	Peru	China	Philippines	India
Cost of treatment per month, US$ [source]								
Cost of BR (intensive)		141 [20, 25]	273 [18]	167 [25, 36]	149 [22]	78 [23]	134 [25]	95 [37]
Cost of BR (continuation)		78 [20;25]	183 [18]	49 [25, 36]	71 [22]	26 [23]	88 [25]	40 [37]
Cost of monitoring per month, US$ [source]								
Bedaquiline + BR	Intensive phase	109 [20]	122 [19]	81 [26]	81 [21]	63 [23]	65^b^	44 [38, 39]
	Continuation phase	98 [20]	94 [19]	64 [26]	70 [21]	55 [23]	595^b^	36 [38, 39]
BR only	Intensive phase	101 [20]	100 [19]	65 [26]	68 [21]	60 [23]	59^b^	43 [38, 39]
	Continuation phase	90 [20]	72 [19]	48 [26]	57 [21]	52 [23]	53^b^	35 [38, 39]
Cost of inpatient and outpatient care, US$ [source]								
Cost of hospitalization for initial MDR-TB per day		268 [24]	231 [18]	84 [26]	55 [21]	38 [24]	20 [24]	16 [30]
Cost of outpatient care for initial MDR-TB	No. of consultation per month (mean)/unit cost	1/41 [24]	1/36 [24]	1/17 [26]	1/11 [21]	1/9 [24]	1/6 [24]	1/3 [30]
	No. of TB nurse home visits per month (mean)/unit cost	28/1 [24]	28/0 [24]	28/NA [26]	28/NA [21]	28/NA [24]	28/NA [24]	28/NA [30]
Disability weights (mean) by health state for all countries analyzed								
Active TB		0.331 [16]						
Culture converted TB (first year)		0.170 [16]						
Culture converted TB (subsequent year)		0.170 [16]						
Lost to follow-up		0.331 (Assumed equal to the active MDR-TB state)						
Treatment complete and cured		0 (Assumed equal to the general population [41])						
Death		1						

*BR* background regimen, *MDR-TB* multidrug-resistant tuberculosis, *NA* Not available, *US* United States

^a^All costs have been converted and inflated to 2013 US$

^b^SOURCE: local hospital

### Health state outcomes

The primary patient outcomes assessed were DALYs averted. The percentage of patients with cure (defined as five consecutive negative cultures from samples collected at least 30 days apart in the final 12 months of treatment) [13], and the number of patients who acquired additional resistance (acquisition of XDR-TB) were also calculated. The primary cost outcomes consisted of direct healthcare costs only, and excluded treatment acquisition costs.

### Price threshold analysis

According to WHO recommendations, three times the *per capita* gross domestic product (GDP) is the threshold cost per DALY where an intervention can be considered cost-effective [30]. Threshold analysis was conducted to determine the price range at which the addition of bedaquiline to BR would be cost-effective in each country setting at both one times the per capita GDP and three times the per capita GDP.

In addition, a probabilistic sensitivity analysis (PSA) was conducted to assess the likelihood of bedaquiline plus BR being cost-effective versus BR alone at prices ranging from 50% of the lowest price at which the drug would meet the minimum standard for the WHO CE threshold, to double the highest price. Details of the PSA and the probabilistic distributions can be found in Additional file 2: Table S2.

## Results

### Base-case clinical outcomes

Over a 10-year time horizon, treatment with bedaquiline plus BR resulted in higher DALYs averted, compared with BR alone. The highest incremental change in DALYs was observed in China where the introduction of bedaquiline to the BR was associated with just 8.87 DALYs per patient compared to 11.86 DALYs per patient with BR alone. This represents a 25.15% reduction in DALYs per patient (Table 3).

**Table 3:** Total and incremental results (bedaquiline plus BR versus BR) in the high burden countries analyzed

	Estonia	Russia	South Africa	Peru	China	Philippines	India
Cohort							
Population^a^, cases (n)	38	6,537	6,494	564	826	11	20,763
Estimated Total DALYs, avoided							
Bedaquiline + BR	438.41	72,824.26	78,816.95	8,318.51	7,329.94	152.50	299,598.12
BR only	554.29	90,479.07	96,530.04	10,475.26	9,792.45	177.64	384,990.40
Estimated DALYs per patient, avoided							
Bedaquiline + BR	11.54	11.14	12.14	14.75	8.87	13.86	14.43
BR only	14.59	13.84	14.86	18.57	11.86	16.15	18.54
Incremental change in DALYs (bedaquiline + BR vs. BR) (%, relative to bedaquiline)	-20.90	-19.51	-18.35	-20.59	-25.15	-14.16	-22.18
Patients with successful outcomes (%)							
Bedaquiline + BR	40.66	38.67	36.50	40.09	47.25	28.69	42.76
BR only	26.29	24.83	23.55	25.88	31.16	17.85	27.97
Incremental change in successful outcomes (bedaquiline + BR vs. BR) (%, relative to bedaquiline)	+54.67	+55.78	+55.02	+54.87	+51.62	+60.78	+52.87
Number of cases of acquired resistance							
Bedaquiline + BR	2.16	349.72	28.20	31.88	52.90	0.50	1,274.00
BR only	3.16	512.37	47.58	46.63	77.04	0.73	1,862.89
Incremental change in acquired resistance (bedaquiline + BR vs. BR) (%, relative to bedaquiline)	-31.59	-31.75	-40.8	-31.62	-31.34	-32.22	-31.61

*BR* background regimen, *DALY* disability-adjusted life years

^a^Population cohort: Russia, China, Philippines: New lab-confirmed MDR-TB cases in 2012 [31]; South Africa: Total number of patients started on MDR-TB treatment in 2012 [31]; Estonia, Peru: laboratory-confirmed MDR-TB cases in 2012 [31]; India: Total number of patients started on MDR-TB treatment in 2013 [2]

Addition of bedaquiline to the BR resulted in a higher percentage of patients experiencing successful outcomes (cured or treatment completed) compared with BR alone. Incremental change in successful outcomes ranged from 51.62% in China (where the introduction of bedaquiline was associated with an increase in successful outcomes from 31.16% to 47.25%) to 60.78% in the Philippines (where the introduction of bedaquiline was associated with an increase in successful outcomes from 17.85% to 28.69%) (Table 3).

In the high- and upper middle-income countries (Estonia, Russia, South Africa, Peru and China), between 31.34% and 40.80% fewer cases of acquired resistance were observed when bedaquiline was added to the BR compared with BR alone. China saw a 31.34% reduction, with 52.90 cases of acquired resistance when bedaquiline was added to the BR, versus 77.04 with BR alone. South Africa saw a 40.8% reduction, with 28.20 cases of acquired resistance when bedaquiline was added to the BR, versus 47.58 with BR alone (Table 3).

In the lower-middle income countries (India and the Philippines), the introduction of Bedaquiline to the BR was also associated with fewer cases of acquired resistance. India saw a reduction of 31.61%, with 1,274 cases of acquired resistance when bedaquiline was added to the BR, versus 1862.89 with BR alone (Table 3). The Philippines saw a reduction of 32.22%, with 0.5 cases of acquired resistance when bedaquiline was added to the BR, versus 0.73 with the BR alone.

A sensitivity analysis evaluating the outcomes of treatment with bedaquiline in a cohort of XDR-TB patients only, demonstrated that bedaquiline was associated with greater DALYs averted in this patient group (Additional file 3: Table S3).

### Cost analysis

Although outpatient care and monitoring costs were increased when bedaquiline was added to the BR compared with BR alone, reductions in hospitalization costs were observed in all settings, resulting in total healthcare cost offsets compared with BR alone (Table 4). Addition of bedaquiline to BR resulted in savings to healthcare costs compared with BR alone in all countries analyzed, with the highest impact expected in Russia (US$194 million) and South Africa (US$43 million) (Table 4).

**Table 4:** Total healthcare costs (excluding treatment acquisition) for bedaquiline plus BR versus BR in the analyzed countries

	Estonia	Russia	South Africa	Peru	China	Philippines	India
Cohort							
Population^a^, cases (n)	38	6,537	6,494	564	826	11	20,763
Hospitalization costs (at 100% hospitalization), US$ total^b^/per patient^c^							
Bedaquiline + BR	2,515,318/66,193	400,933,524/61,333	85,584,735/13,179	8,505,448/15,080	7,792,452/9,434	35,660/3,242	4,892,107/236
BR only	3,780,243/99,480	596,419,985/91,238	129,930,717/20,008	12,699,038/22,516	11,177,776/13,532	52,799/4,800	7,376,327/355
Outpatient care, US$ total^b^/per patient^c^							
Bedaquiline + BR	23,858/628	1,716,115/263	1,221,043/188	47,999/85	77,975/94	394/36	1,740,459/84
BR only	17,667/465	1,109,759/170	1,144 148/20,008	31,283/55	72,985/88	249/23	1,979,948/95
Monitoring costs, US$ total^b^/per patient^c^							
Bedaquiline + BR	66,887/1,760	11,449,710/1,752	7,778,834/1,198	721,800/1,280	930,012/1,126	9,182/835	15,202,670/732
BR only	69,860/1,838	10,165,718/1,555	6,808,367/1,048	682,575/1,210	1,019,445/1,234	8,989/817	16,650,529/802
Total healthcare costs (excluding treatment acquisition costs), US$ total^b^/per patient^c^							
Bedaquiline + BR	2,606,062/68,581	414,099,348/63,347	94,584,612/14,565	9,275,248/16,445	8,800,439/10,654	45,236/4,112	21,835,236/1,052
BR only	3,867,769/101,783	607,695,462/92,962	137,883,232/21,232	13,412,896/23,782	12,270,205/14,855	62,037/5,640	26,006,804/1,253
Incremental health care cost-savings (excluding acquisition costs) (bedaquiline + BR vs. BR), US$ total^b^/per patient^c^	1,261,707/33,202	193,596,114/29,615	43,298,620/6,667	4,137,648/7,337	3,469,766/4,201	16,801/1,528	4,171,568/201

*BR* background regimen, *TB* tuberculosis, *US* United States

^a^Population cohort: Russia, China, Philippines: New lab-confirmed MDR-TB cases in 2012 [31]; South Africa: Total number of patients started on MDR-TB treatment in 2012 [31]; Estonia, Peru: laboratory-confirmed MDR-TB cases in 2012 [31]; India: Total number of patients started on MDR-TB treatment in 2013 [2]; ^b^Total costs (inflated to US$ 2013) reflect costs for the entire cohort of patients; ^c^per patient costs (inflated to US$ 2013) are calculated by dividing the total costs by the population cases (n)

### Price threshold analysis

**Fig. 2:**
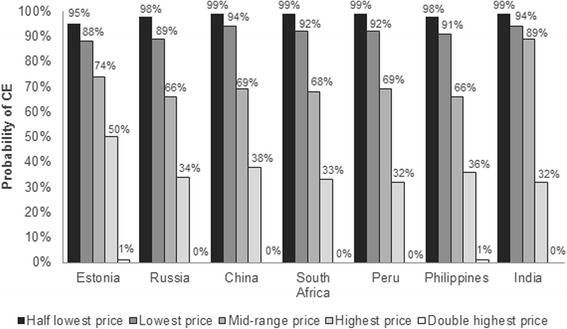
Probability that bedaquiline plus BR will be cost-effective compared with BR alone. conducted to assess whether the addition of bedaquiline to BR, for a range of different prices that satisfy the WHO CE threshold (3x GDP), would be cost-effective. BR: background regimen; CE: cost-effectiveness; GDP: gross domestic product; WHO: World Health Organization

The price per regimen of bedaquiline for which bedaquiline would be cost-effective (according to the WHO criteria of 3 times GDP per capita) in Estonia, Russia, Peru, and China ranged between US$23,904-US$203,492. The range for South Africa was lower at US$29,151-US$72,701, while the Philippines and India demonstrated a lower range, at US$6,996-US$20,323 (Table 5). This is reflective of the low hospitalization costs and the low willingness-to-pay per DALY in these countries. At these prices, the probability that bedaquiline would be cost-effective at thresholds of 3 times GDP per capita (based upon cost per DALY) ranged from 32% to 94% (Fig. 2).

**Table 5:** Range of bedaquiline prices and CE analysis based on these ranges

Bedaquiline price threshold analysis^a^							
	Estonia	Russia	South Africa	Peru	China	Philippines	India
WHO CE thresholds (1x GDP-3x GDP), US$ (Cost per DALY averted)	16,844 – 50,532	14,037 – 42,111	7,352 – 22,056	6,796 – 20,388	6,091 - 18,273	2,587 – 7,761	1,503 – 4,509
Cost-effective price range, US$^b^	91,984 – 203,492	73,909 – 156,427	29,151 – 72,701	36,421 – 92,953	23,904 – 62,593	8,567 – 22,992	6,996 – 20,323
Bedaquiline plus BR vs. BR alone price range for cost-effectiveness analysis							
Half lowest range price, US$	45,992	36,955	14,576	18,211	11,952	4,284	3,498
Lowest range price, US$	91,984	73,909	29,151	36,421	23,904	8,567	6,996
Mid-range price, US$	147,738	115,168	50,926	64,687	43,428	15,285	13,660
Highest-range price, US$	203,492	156,427	72,701	92,953	62,593	22,002	20,323
Double highest range price, US$	406,984	312,854	145,402	185,906	125,186	44,004	40,646

*BR* background regimen, *CE* cost-effectiveness, *DALY* disability-adjusted life years, *GDP* gross domestic product, *US* United States, *WHO* World Health Organization

^a^Price ranges were chosen in order to satisfy the WHO CE threshold (up to 3x GDP of each country)

^b^Per 6-month regimen

## Discussion

Management of MDR-TB is costly to national TB programs. In 122 mostly low- and middle-income countries, accounting for 95% of the global TB burden including MDR-TB, total spending for TB prevention, diagnosis, and treatment increased from US$3.3 billion in 2006 to US$6.3 billion in 2014 (US$3.8 billion for drug-susceptible TB and US$1.8 billion for MDR-TB); the estimated required funding for 2015 is US$8 billion [2]. Costs of managing patients who develop XDR-TB are even higher, as hospital stays and drug costs greatly increase [31]. Our study shows that the addition of bedaquiline to a BR decreased the DALY burden compared with BR alone. This study demonstrated that addition of bedaquiline to a BR reduced cases of acquired resistance. The current model did not account for potential increases in the number of people being treated due to improvements in diagnostic modalities such as molecular diagnostic platforms like the Xpert ® MTB/Rif and line probe assays (which can detect drug resistance within 2 days) that have been introduced in the field of MDR-TB [3]. Faster and more successful treatment of primary cases could reduce secondary transmission and decrease the number of new cases of MDR-TB.

The mortality imbalance observed in the C208 trial was unexplained. Subsequent analysis by an independent investigator determined that there was no causal relationship with bedaquiline and a missing = failure analysis used within the study accounted for these deaths, considering them as treatment ‘failures’[12]. In addition, recent data published based on early access (compassionate use) of bedaquiline, suggests a lower mortality rate than has been observed in clinical trials. In a retrospective cohort study of 35 patients treated with bedaquiline in France, only one patient died (3%), and the death was considered unrelated to TB or TB treatment by the investigator [42]. Similarly, an interim analysis of 91 patients treated with bedaquiline in South Africa reported 3 deaths (3.3%), none of which were considered related to bedaquiline by the investigator [43]. Therefore, although mortality was included in the model, no mortality difference was assumed between bedaquiline and BR treatment arms. Although the increased mortality observed in the bedaquiline arm of study C208 is not believed to be related to bedaquiline [12, 42, 43], we nonetheless conducted a scenario analysis to test how the inclusion of the death would influence the change in DALY. Results show that if the mortality rates from study C208 are separately accounted for in the analysis (instead of lumping the deaths with all those failing treatment, as was done in the original analysis), the number of DALY per patient is likely to increase as a result of the use of bedaquiline (Additional file 3: Table S3). The likely impact of sputum culture conversion in reducing infectiousness of patients with TB and the potential to reduce secondary MDR-TB cases is not accounted for. When transmission rates were included in sensitivity analyses, bedaquiline was associated with additional healthcare cost savings associated with the reduced number of cases (data not shown).

Increases in outpatient care costs observed with the addition of bedaquiline reflected increases in monitoring costs associated with the implementation of any new treatment. Addition of bedaquiline to a BR has been shown to result in faster and higher culture conversion rates compared with BR alone [13], an outcome that is likely to result in decreased need for hospitalization. MDR-TB guidelines in resource-rich settings typically require that patients be hospitalized during treatment [32, 40]; however, the rate of hospitalization in low-to-middle income countries tends to be low [8] and there is a push in such settings to drive community-based treatment [33]. In low-to-middle income countries where the majority of MDR-TB patients are managed in community-based settings, the price at which it is cost-effective to add bedaquiline to a BR may be very low, and may be lower than the price thresholds suggested in this analysis.

The current study expands on the results of a prior study conducted on behalf of the WHO, by using a comprehensive Markov state transition model over a longer time horizon (10 years, compared with 20 months in the WHO analysis), and incorporating more detailed transitions and events such as patient-level data to inform the state transitions, as well as covering additional countries [7]. The WHO analysis estimated that bedaquiline was likely to be cost-effective and cost-saving in Peru, Russia, Estonia, the Philippines, and China, where treatment and management costs are moderate to high. In low-income countries with low drug costs and hospitalization, such as Nepal, addition of bedaquiline to the treatment regimen was likely to not be cost-effective [7], whereas in lower middle-income settings in the current study (the Philippines and India), there was a 32%94% probability that bedaquiline would be cost-effective (in terms of cost per DALY). In high-income settings, such as the UK, previous studies have shown the addition of bedaquiline to BR to be cost-effective, when measuring cost-utilities such as QALYs gained, and DALYs avoided [9].

The price threshold analysis shown in this study suggests that prices higher than the price of bedaquiline in a high income setting, such as the United States, would meet WHO criteria for cost-effectiveness in several of the countries analyzed. While these prices may be cost-effective, many would raise the question as to whether or not such prices are affordable; in Table 4, the cost offsets (mostly attributable to savings from reduced hospitalization) show that in Estonia and Russia, approximately US$30,000 per patient will be saved with the use of bedaquiline; in South Africa, Peru, and China, between US$4,201-US$7,337 per patient; and the Philippines and India, between US$70-US$1,528. Prices that capture at least the cost-offsets are not only cost-effective, but arguably, affordable as long as they result in no net change in health care spending. While cost-effectiveness is an important measure to consider when evaluating new health interventions, budget impact and value for innovation for the first new treatment for tuberculosis in 40 years must also be taken into consideration.

The key strengths of the current study include its comprehensive health state structure, the use of patient-level data to inform state transitions, and the extent to which the model captures WHO guidance on treatment strategies for MDR-TB.

A limitation of the current analysis was that the clinical data were based on the multinational Phase II study for bedaquiline [13] and thus may not reflect local variations in treatment success rates. The study also used UK specific life tables to calculate DALYs as country specific life table data were difficult to source. If the UK life tables are not reflective of the country specific life tables, this could lead to over/underestimation of the DALY burden in each country setting. Other limitations of this study include the fact that possible increases in mortality due to treatment with bedaquiline have been excluded, as well as a lack of empirical data on the price of bedaquiline in the evaluated countries at the time of the analysis.

## Conclusions

Treatment with bedaquiline plus BR is expected to improve health outcomes through reduced DALY burden compared with BR alone. Addition of bedaquiline to BR resulted in total healthcare cost offsets (excluding treatment acquisition costs). At prices required to satisfy the WHO CE threshold, the probability that bedaquiline would be cost-effective was 32%–94% in the high burden countries analyzed.

## Additional files

Additional file 1: Table S1. Summary of epidemiological data, economic profile, and MDR-TB interventions in high burden countries analyzed. (DOCX 64 kb)
Additional file 2: Table S2. Probabilistic analysis parameters and distributions. (DOCX 59 kb)
Additional file 3: Table S3. Scenario analyses. (DOCX 62 kb)

## Abbreviations

BR: Background regimen
CE: Cost-effectiveness
DALY: Disability-adjusted life year
EQ-5D: EuroQoL five dimensions
GDP: Gross domestic product
HR: Hazard ratio
MDR-TB: Multidrug resistant-tuberculosis
NA: Not available
PSA: Probabilistic sensitivity analysis
QALY: Quality-adjusted life year
SE: Standard error
TB: Tuberculosis
UK: United Kingdom
US: United States
WHO: World Health Organization
XDR-TB: Extensively drug-resistant tuberculosis
