# Supplementary material for: Health outcomes of bedaquiline in the treatment of multidrug-resistant tuberculosis in selected high burden countries
Source: BMC Health Serv Res. 2017 Jan 26;17:87. doi: 10.1186/s12913-016-1931-3 (PMC5267460; doi:10.1186/s12913-016-1931-3)
Supplement: Additional file 1: Table S1. — Summary of epidemiological data, economic profile, and MDR-TB interventions in high burden countries analyzed. (DOCX 64 kb) [file 12913_2016_1931_MOESM1_ESM.docx]

Table S1: Summary of epidemiological data, economic profile, and MDR-TB interventions in high burden countries analyzed

|  | Estonia | Russia | South Africa | Peru | China | Philippines | India |
| --- | --- | --- | --- | --- | --- | --- | --- |
| Estimated number of tested MDR-TB cases in 2013 (n) | 222 | 43,743 | 258,401 | 20,459 | 41,812 | 17,241 | 248,341 |
| Number of lab-confirmed MDR-TB cases in 2013 (n) | 54 | 13,521 | 26,023 | 1,462 | 4,183 | 3,962 | 35,385 |
| Number of MDR-TB cases started on treatment in 2013 (n) | 40 | 20,372 | 10,663 | 1,425 | 2,184 | 2,262 | 20,763 |
| GDP/capita in 2012, US$ (income level) | 16,844  (high) | 14,037  (high) | 7,352  (upper middle) | 6,796  (upper middle) | 6,091  (upper middle) | 2,587  (lower middle) | 1,503  (lower middle) |
| Anti-MDR-TB regimen | Individualized | Individualized | Standardized | Standardized | Standardized | Individualized | Unknown |
| Location of treatment | Hospital ward, health clinic | Hospital ward, health clinic | Hospital and community | Clinic | Hospital and clinic | Clinic and patients’ home | Unknown |
| Percentage of patients with MDR-TB/XDR-TB (%) | 92.7 / 7.3 | 88.7 / 11.3 | 89.4 / 10.6 | 93.5 / 6.6 | 92.8 / 7.2 | 99.2 / 0.8 | 96.3 / 3.7 |

SOURCE: [1;2;3;4;5]

GDP: gross domestic product; MDR-TB: multidrug-resistant tuberculosis; US: United States

NOTE: Information included in this table is general and does not reflect inputs to the model

**References**

1. WHO. Global Tuberculosis Report. 2014.

2. WHO, Vassall A. Cost-effectiveness of introducing Bedaquiline in MDR-TB regimens - An exploratory analysis. 2013 Jan 26.

3. Fitzpatrick C, Floyd K. A systematic review of the cost and cost effectiveness of treatment for multidrug-resistant tuberculosis. Pharmacoeconomics. 2012;30(1):63–80.

4. Schnippel K, Rosen S, Shearer K, Martinson N, Long L, Sanne I, et al. Costs of inpatient treatment for multi-drug-resistant tuberculosis in South Africa. Trop Med Int Health. 2013;18(1):109–16.

5. The World Bank Group. GDP per capita (current US$). <http://data.worldbank>. org/indicator/NY.GDP.PCAP.CD 1/ . 2014. 13-1-2015. Ref Type: Online Source
