# Supplementary material for: Health outcomes of bedaquiline in the treatment of multidrug-resistant tuberculosis in selected high burden countries
Source: BMC Health Serv Res. 2017 Jan 26;17:87. doi: 10.1186/s12913-016-1931-3 (PMC5267460; doi:10.1186/s12913-016-1931-3)
Supplement: Additional file 2: Table S2. — Probabilistic analysis parameters and distributions. (DOCX 59 kb) [file 12913_2016_1931_MOESM2_ESM.docx]

Table S2: Probabilistic analysis parameters and distributions

| Parameter | Distribution | Comment |
| --- | --- | --- |
| **Shape and scale parameters for parametric curve used to model baseline (BR only) sputum conversion rates** | Multivariate log-normal | A log-normal distribution is bounded between 0 and infinity, and skewed. A multivariate distribution was used to take into account the correlation between the shape, scale, and treatment parameter outputted by the patient level analysis |
| **Hazard ratio on sputum culture conversion** | Log-normal | Bounded between 0 and infinity, and skewed |
| **Hazard ratio on relapse** | Log-normal | Bounded between 0 and infinity, and skewed |
| **Hazard ratio on culture conversion for subsequent MDR-TB and XDR-TB compared to MDR-TB patients** | Log-normal | Bounded between 0 and infinity, and skewed |
| **Probability of withdrawal** | Beta | Bounded between 0 and 1 |
| **Probability of peripheral neuropathy (linezolid only)** | Beta | Bounded between 0 and 1 |
| **Probability of becoming lost to follow-up** | Beta | Bounded between 0 and 1 |
| **Probability of relapse** | Beta | Bounded between 0 and 1 |
| **Probability of reoccurrence** | Beta | Bounded between 0 and 1 |
| **Percentage of failures leading to additional resistance** | Beta | Bounded between 0 and 1 |
| **Hazard ratio on mortality, MDR-TB, and XDR-TB patients** | Gamma | Bounded between 0 and infinity, and skewed |
| **Probability of mortality** | Beta | Bounded between 0 and 1 |
| **Number of transmitted cases per primary case** | Gamma | Bounded between 0 and infinity, and skewed |
| **Cost of hospitalization** | Log-normal | Bounded between 0 and infinity, and skewed |
| **Percentage of patients receiving different BR treatments** | Beta | Bounded between 0 and 1 |
| **Cost of end of life care (palliative care)** | Gamma | Bounded between 0 and infinity, and skewed |
| **Disability weights for patients with TB** | Beta | Bounded between 0 and 1 |

BR: background regimen; MDR: multidrug-resistant; XDR-TB: extensively drug-resistant tuberculosis
