# Supplementary material for: Health outcomes of bedaquiline in the treatment of multidrug-resistant tuberculosis in selected high burden countries
Source: BMC Health Serv Res. 2017 Jan 26;17:87. doi: 10.1186/s12913-016-1931-3 (PMC5267460; doi:10.1186/s12913-016-1931-3)
Supplement: Additional file 3: Table S3. — Scenario analyses. (DOCX 62 kb) [file 12913_2016_1931_MOESM3_ESM.docx]

Table S3: Scenario analyses

| Outcome | | Estonia | Russia | South Africa | Peru | China | Philippines | | India |
| --- | --- | --- | --- | --- | --- | --- | --- | --- | --- |
| **Scenario 1: XDR-TB only** | | | | | | | | | |
| **Incremental change in DALYs per patient** | | -2.50 | -0.70 | -2.25 | -3.12 | -2.51 | -1.75 | | -3.39 |
| **Scenario 2: Hospitalization increased by 10%** | | | | | | | | | |
| **Incremental health care cost-savings (excluding acquisition costs) (bedaquiline + BR vs. BR), US$ total**/per patient*****  **(base case)** | 1,310,193 / 34,479  (1,261,707 /33,202) | | n/a  (193,596,114 / 29,615) | 43,613,344 / 6,716  (43,298,620 / 6,667) | n/a  (4,137,648 / 7,337) | n/a  (3,469,766 / 4,201) | | n/a  (16,801 / 1,528) | 4,417,366 / 213  (4,171,568 / 201) |
| **Scenario 3: Hospitalization decreased by 10%** | | | | | | | | | |
| **Incremental health care cost-savings (excluding acquisition costs) bedaquiline + BR vs. BR, US$ total**/per patient*****  **(base case)** | | 1,136,475/ 29,907  (1,261,707 /33,202) | 174,156,897 / 26,642  (193,596,114 / 29,615) | 38,895,142 / 5,989  (43,298,620 / 6,667) | 3,721,415 / 6,598  (4,137,648 / 7,337) | 3,134,041 / 3794  (3,469,766 / 4,201) | | 15,108 / 1,373.49  (16,801 / 1,528) | 3,925,770 / 189  (4,171,568 / 201) |
| **Scenario 4: Including mortality imbalance from the C208 trial** | | | | | | | | | |
| **Incremental change in DALYs per patient** | | 1.44 | 1.72 | 1.28 | 1.84 | 1.52 | | 0.89 | 2.21 |

DALY: disability-adjusted life years; XDR-TB: extensively drug-resistant tuberculosis
